# Effects of Chung-Pae Inhalation Therapy on a Mouse Model of Chronic Obstructive Pulmonary Disease

**DOI:** 10.1155/2015/461295

**Published:** 2015-10-11

**Authors:** Joon-Ho Hwang, Beom-Joon Lee, Hee Jae Jung, Kwan-Il Kim, Jun-Yong Choi, Myungsoo Joo, Sung-Ki Jung

**Affiliations:** ^1^Division of Allergy, Immune and Respiratory System, Department of Internal Medicine, College of Korean Medicine, Kyung Hee University, Seoul 130-701, Republic of Korea; ^2^School of Korean Medicine, Pusan National University, Yangsan 626-870, Republic of Korea

## Abstract

Chung-pae (CP) inhalation therapy is a method frequently used in Korea to treat lung disease, especially chronic obstructive pulmonary disease (COPD). This study investigated the effects of CP inhalation on a COPD animal model. C57BL/6 mice received porcine pancreatic elastase (PPE) and lipopolysaccharide (LPS) alternately three times for 3 weeks to induce COPD. Then, CP (5 or 20 mg/kg) was administered every 2 h after the final LPS administration. The effect of CP was evaluated by bronchoalveolar lavage (BAL) fluid analysis, histological analysis of lung tissue, and reverse transcription polymerase chain reaction analysis of mRNA of interleukin- (IL-) 1*β*, tumor necrosis factor- (TNF-) *α*, IL-6, and tumor growth factor- (TGF-) *β*. Intratracheal CP administration reduced the number of leukocytes and neutrophils in BAL fluid, inhibited the histological appearance of lung damage, and decreased the mRNA levels of the proinflammatory cytokines IL-1*β*, TNF-*α*, IL-6, and TGF-*β*. Intratracheal CP administration effectively decreased the chronic inflammation and pathological changes in a PPE- and LPS-induced COPD mouse model. Therefore, we suggest that CP is a promising strategy for COPD.

## 1. Introduction

Inhalation therapy is a treatment technique for administering a variety of inhalable drugs to target lung tissue, airway secretion components, and microorganisms in the upper, central, and/or peripheral airways [1]. Such therapy is used widely to treat chronic obstructive pulmonary disease (COPD) in the respiratory tract [2]. Inhalation administration has an advantage over oral administration for treating respiratory disease in that it allows rapid and substantial drug absorption and has fewer side effects [3]. Typically, herbal medicines are administered orally in the form of decoction or granular extract; however, several studies have reported the direct delivery of herbal medicine to the airway via inhalation [4–7]. The current study employed the MicroSprayer, which generates a plume of liquid aerosol (mass median diameter (MMD) of 16–22 *μ*m), enabling the administration of drugs directly to the lung via the trachea [8].

Chung-pae (CP), composed of* Ephedrae Herba*,* Caryophylli Flos*,* Pogostemonis (Agastachis) Herba*, and* Zingiberis Rhizoma Crudus*, is a representative aerosol agent used in the respiratory clinic at Kyung Hee Oriental Medicine Hospital, Seoul, Korea, for relieving the symptoms of patients with dyspnea and cough. Previously, we investigated the effect of intratracheal (i.t.) CP administration on lipopolysaccharide- (LPS-) induced acute lung injury (ALI) in a mouse model. We found that CP suppressed neutrophil infiltration to the lung and reduced the production of proinflammatory cytokines via decreased expression of the proinflammatory transcription factor, nuclear factor kappa-light-chain-enhancer of activated B cells (NF-*κ*B), and activation of the anti-inflammatory factor, nuclear factor erythroid 2 (NF-E2)-related factor 2 (Nrf2) [7].

The current study investigated the activities of CP on chronic lung injuries including COPD. COPD was selected for the study because it is a prevalent chronic respiratory disease that has become a major public health problem [9]. LPS and porcine pancreatic elastase (PPE) were used to induce COPD in a mouse model. Long-term administration of LPS by inhalation induces emphysema [10–12], and PPE augments the emphysematous changes that are critical characteristics of COPD [13, 14].

In the present study, the effect of CP on chronic lung injury was evaluated in a mouse model of COPD generated using LPS and PPE. The effect of CP was assessed by analyzing bronchoalveolar lavage (BAL) fluid, lung histology, and mRNA levels of proinflammatory cytokines.

## 2. Materials and Methods

### 2.1. Preparation of Chung-Pae Water Extract (CP)

CP was prepared as described previously [7]. Briefly, 20.0 g Ephedrae, 20.0 g Ephedrae Herba, (Agastachis) Herba, 10.0 g Caryophylli Flos, and 10.0 g Zingiberis Rhizoma Crudus were boiled in 1 L distilled water for 2 h. The mixture was concentrated to 50 mL with a low-pressure evaporator and then freeze-dried to yield 6.0 g of powder.

### 2.2. Animals

Male C57BL/6 mice were supplied by Orient Bio Inc. (Seongnam, Korea) and were bred in a pathogen-free facility at Pusan National University, Yangsan, Korea. Animals were housed in certified standard laboratory cages and fed with food and water* ad libitum* prior to the experiments. All experimental procedures were approved by the Guidelines of the Institutional Animal Care and Use Committee of Pusan National University, Busan, Republic of Korea (protocol number: PNU-2010-00028).

### 2.3. COPD Mouse Model and Treatment

COPD was induced in mice using the method reported previously with some modifications [15]. A MicroSprayer (syringe assembly, MSA-250-m, the Penn Century Inc., PA, USA) was used to deliver all materials to the lungs via i.t. Mice (20–30 g) were exposed to 0.25 U of PPE (on days 1, 7, and 14) and 7.0 *μ*g of LPS (on days 4, 11, and 18) for three consecutive weeks. In this manner, the treated mice received PPE and LPS alternately. Two doses of CP (low dose of 5 mg/kg and high dose of 20 mg/kg) in 25 *μ*L of PBS were administered 2 h after every LPS administration. The vehicle-treated group was treated with 25 *μ*L of PBS using the same method and treatment schedule as the CP-treated group. Normal, untreated non-COPD mice were included as a control in the analyses.

### 2.4. BAL Fluid Analysis

BAL fluid analysis was conducted on day 21. BAL was obtained using two consecutive instillations of PBS (1.0 mL) using a 24-gauge intravascular catheter. The total cell number was determined using a hemocytometer. Macrophages, lymphocytes, and neutrophils were counted by Hemacolor (Merck, Darmstadt, Germany) after centrifugation and staining; 100 cells were counted for each microscopic field, and the mean number of cells per field was reported.

### 2.5. Lung Histological Analysis

Mice were perfused with saline and the whole lung was inflated with fixatives. After paraffin embedding, lung tissue were cut in 5-*μ*m thick slices and stained with hematoxylin and eosin (H&E). Three separate H&E-stained sections were evaluated in each mouse under a microscope using 100x magnification.

### 2.6. Isolation of Total RNA from Tissue and Reverse-Transcription-Polymerase Chain Reaction (RT-PCR)

Total RNA was isolated with the QIAGEN RNeasy mini kit (Qiagen, Hilden, Germany) according to the manufacturer's instructions. Two micrograms of total RNA were reverse-transcribed by M-MLV reverse transcriptase (Promega, Madison, WI, USA), and single-stranded cDNA was amplified by PCR using specific primers. The forward and the reverse primers for interleukin- (IL-) 1*β* were 5′-TCATGGGATGATGATGATAACCTGCT-3′ and 5′-CCCATACTTTAGGAAGACACGGATT-3′, respectively; the primers for tumor necrosis factor- (TNF-) *α* were 5′-GGCAGGTCTACTTTGGAGTCATTGC-3′ and 5′-ACATTCGAGGCTCCAGTGAATTCGG-3′, respectively; the primers for IL-6 were 5′-CTGGTGACAACCACGGCCTTCCCTA-3′ and 5′-ATGCTTAGGCATAACGCACTAGGTT-3′, respectively; the primers for tumor growth factor- (TGF-) *β* were 5′-GCGGCAGCTGTACATTGACT-3′ and 5′-ACTGTGTGTCCAGGCTCCAA-3′, respectively; and the primers for glyceraldehyde-3-phosphate dehydrogenase (GAPDH) were 5′-GGAGCCAAAAGGGTCATCAT-3′ and 5′-GTGATGGCATGGACTGTGGT-3′, respectively. For PCR amplification,* Taq*PCRx DNA polymerase recombinant (Invitrogen, Carlsbad, CA, USA) was used according to the manufacturer's protocol. The reaction conditions were as follows: initial denaturation at 95°C for 5 min followed by 22–30 cycles of denaturation for 40 sec at 95°C, annealing for 40 sec at 57°C, and extension for 50 sec at 72°C with a final extension for 7 min at 72°C. Amplicons were separated in 1.2% agarose gels in boric acid buffer at 100 V for 30 min, stained with ethidium bromide, and visualized under UV light. GAPDH was used as an internal control to evaluate the relative expressions of IL-1*β*, TNF-*α*, IL-6, and TGF-*β*.

### 2.7. Statistical Analysis

Group comparisons were performed using one-way analysis of variance (ANOVA) with Duncan's post hoc test. The analysis was conducted using SPSS 18.0 for Windows (SPSS, Chicago, IL, USA). *p* values < 0.05 were considered to indicate significant differences. All experiments were performed independently at least three times.

## 3. Results

### 3.1. Effect of CP on the Total Cell Count and Inflammatory Cell Numbers in the BAL Fluid of PPE- and LPS-Induced COPD Mice

**Figure 1:**
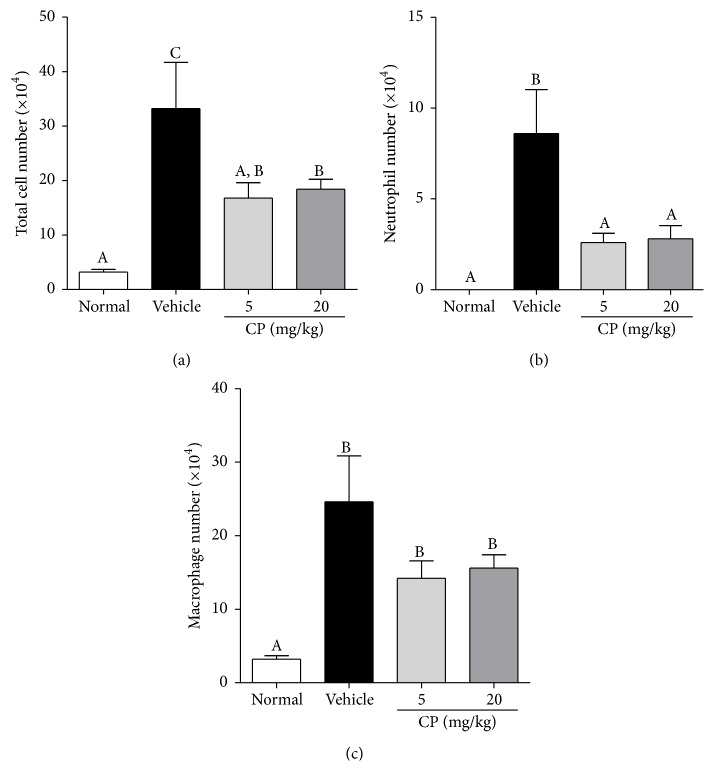
Effect of CP on the total cell number (a), number of neutrophils (b), and number of macrophages (c) in the BAL fluid of PPE- and LPS-induced COPD mice. Data are presented as means ± SEM (*n* = 5). Letters (A–C) indicate different levels of significance (95% level, Duncan's test).

The total cell and neutrophil counts in the BAL fluid of PPE- and LPS-induced COPD mice increased significantly compared to those in the normal group (*p* < 0.01, Figures 1(a) and 1(b)). CP treatment significantly decreased the total cell and neutrophil counts in the BAL fluid compared to the vehicle-treated group (*p* < 0.05, Figures 1(a) and 1(b)). However, no difference was detected between groups treated with 5 or 20 mg/kg CP.

The macrophage population in the vehicle-treated group increased significantly compared to that in the normal group (*p* < 0.01, Figure 1(c)). However, CP did not decrease the macrophage population in the BAL fluid compared to the vehicle-treated group.

### 3.2. Effect of CP on the Histological Evidence of Lung Damage in PPE- and LPS-Induced COPD Mice

**Figure 2:**
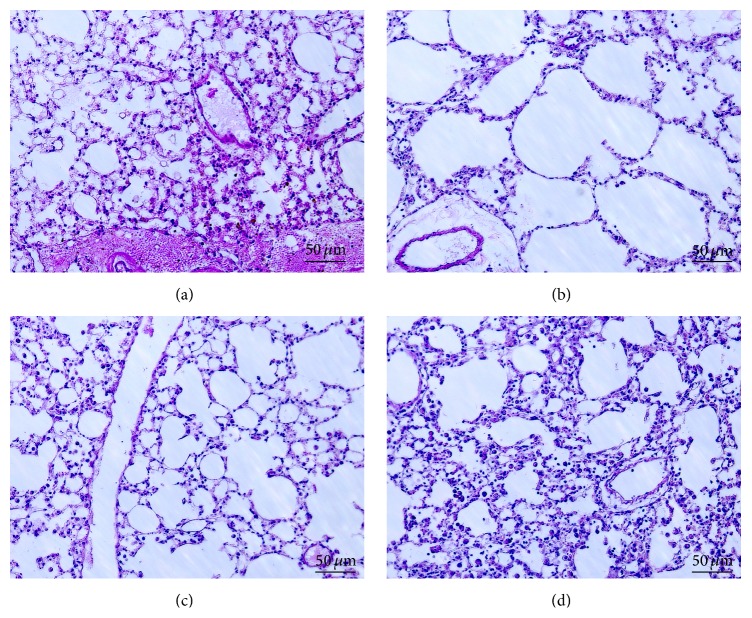
Effect of CP on the histological evidence of lung damage in the PPE- and LPS-induced COPD mice: (a) normal group; (b) vehicle-treated group; (c) CP-treated group (5 mg/kg); and (d) CP-treated group (20 mg/kg).

Larger vacuoles were present in the lung sections of vehicle-treated mice (Figure 2(b)) compared with the normal group (Figure 2(a)). Such enlarged air spaces suggested alveolar destruction due to emphysematous change. However, CP-treated COPD mice showed smaller vacuoles (Figures 2(c) and 2(d)) compared to the vehicle-treated group, suggesting that CP (5 or 20 mg/kg) ameliorated inflammation in the lung.

### 3.3. Effect of CP on the mRNA Expression Levels of Cytokines in the PPE- and LPS-Induced COPD Mice

**Figure 3:**
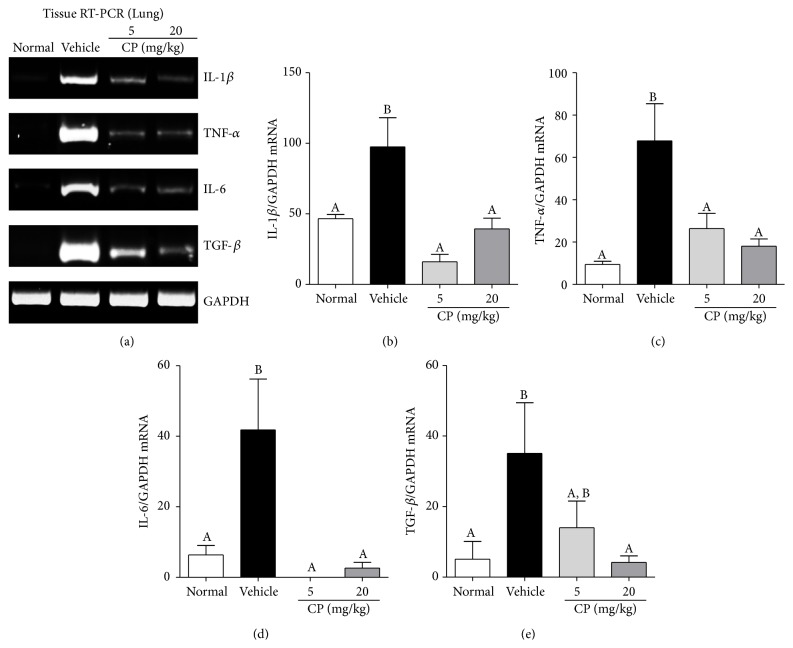
Effect of CP on the mRNA levels of cytokines in the lung of PPE- and LPS-induced COPD mice. Mice were exposed to PPE (on days 1, 7, and 14) and LPS (on days 4, 11, and 18) and administered 5 mg/kg or 20 mg/kg of CP 2 h after every LPS administration. The lungs of variously treated mice were harvested on day 21 for RT-PCR analysis. The intensity of each PCR band was measured by densitometric analysis (a), and relative expression of each gene was calculated over GAPDH. CP reduced the mRNA level of these cytokines (b–e). Data are presented as means ± SEM (*n* = 5). Letters (A–C) indicate different levels of significance (95% level; Duncan's test).

CP (5 or 20 mg/kg) decreased the mRNA levels of IL-1*β*, TNF-*α*, and IL-6 in the lung (Figure 3), which was in agreement with the decreased numbers of inflammatory cells in the BAL fluid. However, a significant decrease in TGF-*β* expression was observed only in mice treated with 20 mg/kg CP.

## 4. Discussion

In the current study, i.t. administration of CP to PPE- and LPS-induced COPD mice reduced the number of leukocytes and neutrophils in the BAL fluid, inhibited lung injury, and decreased the mRNA levels of the proinflammatory cytokines IL-1*β*, TNF-*α*, IL-6, and TGF-*β*.

In our clinic, patients usually received CP at a daily dose of 5 mg/kg; however, long-term administration of 20 mg/kg of CP for 3 weeks showed no adverse effect on vital organs, including the liver and kidney (data not shown). Therefore, in this study, we administered CP at doses of 5 or 20 mg/kg in the PPE- and LPS-induced COPD mice. Infiltration of inflammatory cells in the BAL fluid was observed in the PPE- and LPS-induced COPD mice. Subsequent i.t. administration of CP reduced the total number of infiltrating cells, especially neutrophils, suggesting that CP could inhibit neutrophils, the most deleterious inflammatory mediator in COPD. However, CP did not significantly decrease the macrophage number compared to the vehicle-treated group (42.3% versus 37.6%); this result was consistent with a previous study [7].

COPD is characterized mainly by increased levels of activated neutrophils, macrophages, and T-lymphocytes [16]. Macrophages mediate inflammation in COPD through the release of chemokines that attract neutrophils, monocytes, and T-cells [17]. Neutrophils are key mediators of COPD, as they migrate to the airway under the control of chemotactic factors and become activated [18, 19]. Activated neutrophils secrete proteolytic enzymes that can induce emphysema as well as numerous lung-damaging, proinflammatory cytokines and chemokines (e.g., matrix metalloproteinase- (MMP-) 8, 9, and 12) [20–22]. Moreover, increased numbers of neutrophils in the airway lumen and BAL fluid in individuals with COPD are correlated with disease severity [23, 24].

The COPD model used in this study involved the inhalation of LPS and elastase to induce emphysematous change [14, 25]. Generally, emphysema is induced by a proteolytic-antiproteolytic imbalance. Proteolytic enzymes may augment the inflammatory cell influx into airspaces, which causes destruction of alveolar septa and increased airspaces [10, 26]. Thus, air space enlargement is a criterion used for measuring the severity of emphysematous change [10, 27, 28]. In the current study, CP reduced the vacuole size compared to that in the vehicle group, suggesting that it prevented alveolar destruction. Histological analysis of lung tissue showed increased cell and neutrophil numbers in the BAL fluid.

Numerous cytokines play important roles in the pathological processes of COPD through the recruitment, activation, and survival of inflammatory cells. TNF-*α* and IL-1*β* have long been known to be classical proinflammatory cytokines that contribute to the development of COPD [29–31]. IL-6 is stimulated by TNF-*α* and IL-1*β* and also plays a critical role in the pathogenesis of emphysematous change [32]. These proinflammatory cytokines influence one another and amplify the inflammatory response in COPD [16, 33]. TGF-*β*, a profibrotic cytokine, is one of the main mediators involved in tissue remodeling in the lungs and contributes to architectural changes in the lungs in COPD [34–37]. The blocking of TGF-*β* improves emphysematous changes [38, 39], although a low concentration of activated TGF-*β* is required to maintain alveolar homeostasis and prevent the development of emphysema [40, 41]. Therefore, inhibition of proinflammatory cytokines is one of the most promising treatments for COPD [42]. In this study, CP reduced the mRNA levels of these cytokines in the lung, suggesting the suppression of chronic inflammation and pathological changes as well as the associated neutrophil infiltration in the lung.

## 5. Conclusion

Previously, we have demonstrated the therapeutic effect of i.t. CP administration on ALI [7]. The current study provides experimental evidence that long-term administration of CP has a therapeutic effect on chronic lung injury in a COPD mouse model induced by PPE and LPS. The anti-inflammatory effect exerted by i.t. CP administration suggests that it could be a new therapeutic formula and that inhalation of herbal medicine can be a promising strategy for treatment of COPD.
